# An Alliance of *Trifolium repens*—*Rhizobium leguminosarum* bv. *trifolii*—Mycorrhizal Fungi From an Old Zn-Pb-Cd Rich Waste Heap as a Promising Tripartite System for Phytostabilization of Metal Polluted Soils

**DOI:** 10.3389/fmicb.2022.853407

**Published:** 2022-04-15

**Authors:** Ewa Oleńska, Wanda Małek, Marzena Sujkowska-Rybkowska, Sebastian Szopa, Tadeusz Włostowski, Olgierd Aleksandrowicz, Izabela Swiecicka, Małgorzata Wójcik, Sofie Thijs, Jaco Vangronsveld

**Affiliations:** ^1^Faculty of Biology, University of Bialystok, Bialystok, Poland; ^2^Faculty of Biology and Biotechnology, Institute of Biological Sciences, Maria Curie-Skłodowska University, Lublin, Poland; ^3^Institute of Biology, Warsaw University of Life Sciences-SGGW, Warsaw, Poland; ^4^SHIM-POL A.M. Borzymowski, Izabelin, Poland; ^5^Laboratory of Applied Microbiology, University of Bialystok, Bialystok, Poland; ^6^Environmental Biology, Centre for Environmental Sciences, Hasselt University, Diepenbeek, Belgium

**Keywords:** white clover, plant growth-promoting bacteria (PGPB), nitrogenase, nodule anatomy, metal tolerance, ICP-OES, gas chromatography, transmission electron microscopy

## Abstract

The Bolesław waste heap in South Poland, with total soil Zn concentrations higher than 50,000 mg kg^–1^, 5,000 mg Pb kg^–1^, and 500 mg Cd kg^–1^, is a unique habitat for metallicolous plants, such as *Trifolium repens* L. The purpose of this study was to characterize the association between *T. repens* and its microbial symbionts, i.e., *Rhizobium leguminosarum* bv. *trifolii* and mycorrhizal fungi and to evaluate its applicability for phytostabilization of metal-polluted soils. Rhizobia originating from the nutrient-poor waste heap area showed to be efficient in plant nodulation and nitrogen fixation. They demonstrated not only potential plant growth promotion traits *in vitro*, but they also improved the growth of *T. repens* plants to a similar extent as strains from a non-polluted reference area. Our results revealed that the adaptations of *T. repens* to high Zn-Pb-Cd concentrations are related to the storage of metals predominantly in the roots (excluder strategy) due to nodule apoplast modifications (i.e., thickening and suberization of cell walls, vacuolar storage), and symbiosis with arbuscular mycorrhizal fungi of a substantial genetic diversity. As a result, the rhizobia-mycorrhizal fungi-*T. repens* association appears to be a promising tool for phytostabilization of Zn-Pb-Cd-polluted soils.

## Introduction

Metal pollution of soils is a significant problem worldwide; it undermines the quality and fertility of soils and is an obstacle to sustainable development (Tchounwou et al., 2012). Soils can be natural as well as anthropogenically enriched with trace metals, which can be taken up by plants, and thereafter spread through the food chains (Walker et al., 2012). Metal ions, even at low concentrations, can hamper living organisms by disturbing cell metabolism, damaging the anatomical structure of tissues, leading to growth reduction, accelerated senescence, and necrosis, which may result in a reduction of population size, density, and genetic variability (Jaishankar et al., 2014). Consequently, metal pollution acts as a natural selection power on non-fit organisms and favors the survival and reproduction of individuals that carry adaptive traits (Macnair, 1997).

Certain plants have evolved ways to deal with high metal pollution (Viehweger, 2014; Wójcik et al., 2017; Nikalje and Suprasanna, 2018), and are associated with specific microbial communities that can be valuable in the remediation of polluted areas (Weyens et al., 2009a,b; Thijs and Vangronsveld, 2015; Yan et al., 2020). Metal accumulators possess the ability to store metal ions in aerial tissues (Lange et al., 2017), while excluders retain metals in their roots, thereby preventing metal translocation into the shoots (Shackira and Puthur, 2019; Zgorelec et al., 2020). Microorganisms inhabiting the rhizosphere of such plants can be of significant importance for their application in phytostabilization (Thijs and Vangronsveld, 2015; Wang et al., 2020). The beneficial effects of plant growth-promoting (PGP) bacteria on the metal tolerance of their hosts can be attributed to a lowered external or internal availability of metals due to incorporation of metals in the bacterial cell walls, metal sequestration inside bacterial cells, efflux and precipitation of metal ions on bacterial cell walls, or exopolysaccharide production. PGP bacteria can also indirectly improve plant growth due to traits like enhancing nutrient availability, synthesis of growth hormones, increasing the antioxidative status of plants, and/or protecting the plants from diseases and pathogens (Hayat et al., 2010; Abhilash et al., 2012; Oleńska et al., 2020a). Hence, identification of the most effective plant-microbe association is of significant importance for phytostabilization.

Legumes (*Fabaceae*) are commonly found as pioneer plants on metal-polluted sites, also on waste deposits of metal ore mining and processing in southern Poland (Nowak et al., 2011). They are well suited for site stabilization since they possess extensive root systems to protect soil from erosion, improve aeration for microbial activity, increase soil humus content, synthesize substantial amounts of biomass rich in proteins, and co-exist in symbiosis with rhizobia providing ammonium in the process of atmospheric nitrogen reduction (Padilla and Pugnaire, 2006; Li et al., 2007; Fterich et al., 2014; Roa-Fuentes et al., 2015). In southern Poland, some sites are extremely polluted with metals from the anthropogenic origin, including postindustrial waste deposits, where the soils are additionally highly deficient in water and nutrients (Wójcik et al., 2014). White clover (*Trifolium repens*), a member of the legume family, is found frequently on the more than 100-year-old Zn-Pb waste heap Bolesław in southern Poland (Nowak et al., 2011; Oleńska and Małek, 2013b). *Rhizobium leguminosarum* bv. *trifolii* bacteria were identified as inhabitants of plant root nodules (Oleńska and Małek, 2015), and this partnership might be a promising plant-microbe association for site stabilization. Earlier results (Oleńska and Małek, 2013b) revealed that *R. leguminosarum* bv. *trifolii* of the Bolesław waste heap carries genes whose products are involved in metal ion exclusion, metal tolerance as well as the production of exopolysaccharides with specific sugar composition (Oleńska et al., 2021). Moreover, Oleńska and Małek (2015, 2019) found that *R. leguminosarum* bv. *trifolii* populations inhabiting root nodules of white clover established on the Bolesław Zn-Pb waste heap show a moderate level of genetic diversity, while studies of other European *R. leguminosarum* bv. *trifolii* populations from metal-polluted sites show a drastic reduction of genetic diversity of these populations, which were not able to fix nitrogen (e.g., in Woburn, United Kingdom), and even lack any rhizobial symbionts (e.g., in Braunschweig, Germany) (Chaudri et al., 1993; Giller et al., 1998; Lakzian et al., 2002).

Since the level of genetic polymorphism of bacterial populations stands for adaptability and tolerance of bacteria to changing environmental conditions, *T. repens* nodule microsymbionts from the Bolesław waste heap might be interesting partners for symbiosis. It is worth noting that a symbiosis may also be established between *T. repens* and mycorrhizal fungi (Li et al., 2005). Indeed, endo- or ectomycorrhizal fungi were found to improve the growth of many plant species under metal stress conditions (Luo et al., 2014; Ważny et al., 2021). For example, symbiotic mycorrhizal fungi can sequester metal ions in their hyphae, acting as a barrier toward metals, and thus indirectly protect plant roots. Hence, white clover fitness in metal-polluted areas, such as the Bolesław waste heap, might be increased due to the joint action of rhizobia and mycorrhizal fungi.

In this study, we characterized the *R. leguminosarum* bv. *trifolii*—arbuscular mycorrhizal fungi (AMF)—white clover association as a potential tool in phytostabilization of metal-polluted soils. To investigate the activities of the *T. repens* nodule microsymbionts, *R. leguminosarum* bv. *trifolii* strains were isolated from root nodules of white clover growing on the Bolesław waste heap as well as on the reference grasslands of Bolestraszyce (Przemyskie Fothills). The rhizobial strains were studied for their Zn, Pb, and Cd tolerance, nodulation ability, the polymorphism of *nodA* genes encoding proteins involved in the nodulation process, nitrogenase enzyme activity, as well as for their PGP traits. To determine the effects of *R. leguminosarum* bv. *trifolii* on white clover growth, morphological parameters of plants, as well as biochemical ones of *T. repens* inoculated with the waste heap and reference site rhizobia, were studied. To define the strategy of the plants to deal with metals (accumulator vs. excluder), metal concentrations in leaves and roots were determined. Both light and transmission electron microscopy (TEM) analysis of root nodules were used to evaluate the adaptation of *T. repens* to metals. Soil nutrients (total N and nitrate, ammonia) and micro/macro-element concentrations were determined. The presence of mycorrhiza in white clover roots was investigated using TEM, and the genetic diversity of mycorrhizal populations was estimated using ARISA (automated ribosomal intergenic spacer analysis) fingerprinting.

## Materials and Methods

Five *T. repens* root and leaf samples, soil samples, as well as forty-two *R. leguminosarum* bv. *trifolii* strains that were previously isolated (Oleńska and Małek, 2015) from nodules of *T. repens* that originated from the more than 100-year-old Zn-Pb waste heap in Bolesław (50°17′N 19°29′E, Silesia-Krakow Upland, Poland) and a grassland in the Bolestraszyce (49°48′N 22°50′E, Przemyskie Foothills, Poland) reference area, were used in this study (Supplementary Table 1).

### *Rhizobium leguminosarum* bv. *trifolii* Analysis

#### *Rhizobium leguminosarum* bv. *trifolii* Tolerance to Metals

The studied rhizobial strains were tested for their Zn, Pb, and Cd tolerance on plates with solid a 79CA medium (Oleńska and Małek, 2015) enriched with metal salts, i.e., 0.1, 0.5, and 2.5-mM ZnSO_4_ × 7 H_2_O; 0.1, 0.5, and 1-mM CdCl_2_ × 2.5 H_2_O; 0.1, 0.5, 0.5, 1, and 2.5-mM Pb(NO_3_)_2_ in three replicates (Lakzian et al., 2002, 2007). After 4 days of incubation, positive or negative rhizobial growth results were presented as a binary system (0—no growth, 1—growth).

#### *Rhizobium leguminosarum* bv. *trifolii* Nodulation Ability

The nodulation abilities of the waste heap as well as reference origin *R. leguminosarum* bv. *trifolii* strains were investigated in a laboratory plant test. Commercially certified seeds of *T. repens* cultivar Tasman were sterilized and germinated according to the conditions described by Oleńska and Małek (2015). Two-day-old white clover seedlings were placed in a nitrogen-deficient Hoagland medium in tubes (Oleńska and Małek, 2015), inoculated with the rhizobial strains, and cultivated in a greenhouse for 6 weeks at 19–23°C with a 12/12-h light/darkness cycle. To determine the ability of *R. leguminosarum* bv. *trifolii* to establish a symbiosis with *T. repens*, the presence and the color of root nodules as well as the size and color of plants were estimated in comparison to plants non-inoculated.

#### *Rhizobium leguminosarum* bv. *trifolii* Genetic Diversity of the *nodA* Gene

Genomic DNA was isolated from *R. leguminosarum* bv. *trifolii* strains according to the procedure described by Oleńska and Małek (2015). To amplify the *R. leguminosarum* bv. *trifolii* strains *nodA* gene fragment, the following primers were used: nodA-1 (5′-TGCRGTGGAARNTRNNCTGGGAAA-3′), and nodA-2 (5′-GGNCCGTCRTCRAAWGTCARGTA-3′) (Haukka et al., 1998) under optimized PCR cycling conditions: initial denaturation at 95°C for 15 min, 35 cycles of denaturation at 94°C for 45 s, annealing at 55°C for 45 s, extension at 68°C for 2 min, and final extension at 72°C for 5 min (Ardley et al., 2013). The *nodA* gene sequences of the studied strains, as well as reference ones obtained from the GenBank database (National Center for Biotechnology Information, NCBI), were aligned and inspected using the BioEdit program (Hall, 1999). Phylogenetic analysis of the *nodA* gene, as well as a determination of *R. leguminosarum* bv. *trifolii* strains NodA protein amino acid sequences, were performed using MEGA version 7.0 software (Kumar et al., 2016). Phylogenetic Neighbor Joining tree construction involved an analysis of 1,000 resampled data sets according to the Maximum Composite Likelihood model.

#### *Rhizobium leguminosarum* bv. *trifolii* Nitrogenase Activity

Nitrogenase activity of rhizobia was evaluated using the acetylene reduction assay (ARA) that relies on a gas chromatography monitoring the reduction of acetylene (C_2_H_2_) to ethylene (C_2_H_4_) (Seefeldt et al., 2013; Haskett et al., 2021). For this purpose, from tubes that were tightly closed with rubber caps and containing the inoculated 6-week-old *T. repens* plants growing in a nitrogen-deficient Hoagland medium, 10% (v/v) of the gas phase was replaced with acetylene. After 1-h incubation at room temperature, 1 mL of gas sample was taken from the tubes, injected into the Hewlett Packard GC system (HP 5890 series II, Hewlett Packard, Inc., United States) to determine the ethylene concentration. A 274.3-cm long stainless-steel column packed with Porapak™ Q (80–100 mesh) was used. The temperatures of the injector, column oven, and detector were 150, 230, and 230°C, respectively. Nitrogen of ultrahigh purity was used as a carrier gas. Nitrogenase activity was displayed as ethylene concentration (nMe) nmol⋅h^–1^ calculated on the basis of the percentage of acetylene conversion (% Ac) and the ethylene volume (Ve) using formulas described in Staal et al. (2001).

#### *In vitro* Plant Growth-Promoting Properties of the *Rhizobium leguminosarum* bv. *trifolii* Strains

Beneficial traits of the rhizobia for plants were estimated as: (i) nutrient availability enhancers (production of siderophores and organic acids and solubilization of phosphate), (ii) production of plant growth regulators (IAA and ACCD), and (iii) production of compounds involved in plant disease prevention (acetoin synthesis). Most of the tests were qualitatively, colorimetrically assessed. A phosphate solubilization index (SI) was estimated by plating on a selective NBRIP medium, and IAA production, as well as ACCD activity, was quantitatively assessed. The ability to synthesize organic acids was assessed using Alizarine Red S according to Cunningham and Kuiack (1992). Siderophore production was tested using chrome-azurol S according to Schwyn and Neilands (1987), and a 284 medium (Schlegel et al., 1961). Bacterial acetoin production was examined using the α-naphthol method (Romick and Fleming, 1998). The capacity to solubilize phosphate was tested according to Pikovskaya (1948) with the modifications of Nautiyal (1999), and the phosphate solubilization index (SI) was calculated using an equation described in Pande et al. (2017). The capability of bacteria to synthesize IAA was investigated according to Patten and Glick (2002). Quantification of the synthesized IAA by the rhizobial strains was performed according to Penrose and Glick (2004) using the calibration curve equation (Supplementary Table 1) obtained as a result of the optical density measurements of 0-, 1-, 5-, 10-, 25-, and 45-μg mL^–1^ IAA solutions at a wavelength of 535 nm. Bacterial ACCD activity was studied according to Belimov et al. (2005) and was expressed as α-ketobutyrate concentration and converted into protein concentration. The α-ketobutyrate concentration was estimated using a calibration curve equation (Supplementary Table 1) based on the correlation of the optical density: 0.1-, 0.2-, 0.5-, 0.8-, 1-μM α-ketobutyrate mL^–1^ measured at wavelength of 540 nm. Finally, the ACCD activity was expressed as nM α-ketobutyrate mg^–1^ protein. Protein concentrations were determined according to the method described by Bradford (1976) and calculated on the basis of a calibration curve equation (Supplementary Table 1), where 0.125-, 0.25-, 0.5-, 0.75-, 1.-, 1.5-, 2.-mg protein mL^–1^ of bovine serum albumin (BSA), used as a standard, were measured at a wavelength of 595 nm.

#### Effects of *Rhizobium leguminosarum bv. trifolii* on Growth of White Clover

In order to evaluate the effects of *R. leguminosarum* bv. *trifolii* on *T. repens* growth, the morphological and biochemical parameters of plants were determined after 6 weeks of growth. Plants were inoculated with *R. leguminosarum* bv. *trifolii* strains from the metal-polluted waste heap (WH group) or from the non-polluted reference area rhizobia (R group) (100-μL of an 18-h old liquid bacterial culture adjusted to 0.6 at OD_590_); control plants were not inoculated (NI group). The following morphological parameters were determined: dry and fresh weight of shoots and fresh weight of roots, length of main root, number and length of side roots, total length of the root system, number of nodules and leaves, and ash content.

As biochemical parameters, concentrations of photosynthetic pigments (chlorophyll *a*, *b*, total chlorophyll, and chlorophyll *a* to *b* ratio), and protein concentrations in leaves of *T. repens* were used. Two leaflets (first and third) of a trifoliate leaf of white clover were washed in distilled water, weighted, homogenized in TissueLyser LT (Qiagen), and examined for the photosynthetic pigments according to Wellburn and Lichtenthaler (1984) with modifications described in Wellburn (1994). Photosynthetic pigments concentrations were expressed as μ*g* of a photosynthetic pigment per *g* of fresh weight of plant tissue. Quantitative estimation of proteins was done using the Lowry et al. (1951) method in one leaflet (central) of a trifoliate leaf, and its concentration was expressed as *mg* of proteins *g*^–1^ fresh weight.

### *Trifolium repens* Analysis

#### Root Nodule Anatomy and Arbuscular Mycorrhizal Fungi in Roots

Nodules of different size and stages of development were collected from *T. repens* plants grown on the waste heap in Bolesław and the reference area in Bolestraszyce. The nodules were fixed according to Karnovsky (1965) and Łotocka et al. (1997) for 24-h at 21°C ± 0.5 and air pressure of –0.4-kGcm^–2^. Subsequently, nodules were post-fixated in 1% OsO_4_ for 4-h at 4°C, dehydrated in increasing concentrations of ethanol, and embedded in glycid ether 100 epoxy resin (SERVA) according to Sujkowska-Rybkowska et al. (2012). Blocks were sectioned using microtomes (Jung RM 2065 and Ultracut UCT, Leica). Semithin sections of epoxy resin-embedded nodule tissue blocks were stained with methylene blue and azur A, and examined under a light microscope (Olympus-Provis, Japan). Next, thin sections were collected on copper grids, contrasted with uranyl acetate followed by lead citrate for 1 min, and examined under a TEM Morgagni 268D (Philips, Netherlands).

#### Metal Concentrations in *Trifolium repens* Leaves and Roots

Dry samples of roots and leaves were digested as described earlier (Oleńska et al., 2020b). After digestion, the samples were diluted (Karaś et al., 2021), and an ICPE-9820 (Shimadzu, Kyoto, Japan) with a mini-torch was used for the qualitative and quantitative detection of elements (Zn, Pb, Cd). Prior to analysis, the Sigma-Aldrich (St. Louis, MO, United States) periodic table mix 1 for ICP containing 10-mg L^–1^ of Zn, Pb, and Cd in 10% nitric acid (comprising HF traces) was used for calibration of the ICP-OES (inductively coupled plasma optical emission spectrometry). Simultaneously, analysis was performed for standards. Standard curve equations are given in Supplementary Table 1. Both a negative control (a blanc sample) and a positive control (tomato leaves, NIST^®^ 1573a, Sigma-Aldrich) were included. To preserve the standard/sample conditions, the matrix match method was used.

### Nutrient Concentrations in Soils and Soil Dry Weight

#### Mg, Ca, K, and Na Concentration in Soil

Macroelements were determined by ICP-OES after mineralization of five samples, representative to the Bolesław waste heap and the non-polluted reference grassland, according to the protocol described earlier (Oleńska et al., 2020b). Simultaneously, nutrient contents were determined in a blanc sample, and a standard reference material Montana II soil (NIST^®^ 2711a, Sigma-Aldrich). Soil dry weight was determined according to PN-ISO-11465:1999.

#### Total Kjeldahl Nitrogen

Air-dry soil samples (5-g) were digested with 15-mL concentrated sulfuric acid (H_2_SO_4_) in the presence of 15-g catalyst (96% K_2_SO_4_ and 4% CuSO_4_ × 5 H_2_O) using an automated digestion block Digestor™ 2520 (FOSS) at 420°C for 3-h [Association of Official Analytical Chemist (AOAC), 1990]. In result, nitrogen present in the soil was transformed to ammonium sulfate (NH_4_HSO_4_) (PN-EN 13342:2002, PN-ISO 5664:2002). After cooling, the acid digestion mixtures were distilled by alkalization with 50% NaOH using the auto distillation unit Kjeltec™ 2200 (FOSS) to convert NH_4_^+^ and obtain ammonia NH_3_ gas in solution. To quantify the amount of ammonia in solution, ammonium trapped as ammonium borate in 4% boric acid solution was titrated with 0.01-M HCl in the presence of a Tashiro indicator (0.1-g 100-mL^–1^ bromocresol green and 0.1-g 100-mL^–1^ methyl red in 1-L 95% ethanol). Simultaneously, the digestion, distillation, and titration of the blank sample (sucrose, 1-g) were performed. Quantity of ammonia (TKN determination) was expressed as percentage and calculated according to the formula


$$\%N=\frac{\left(\text{Vx}-\text{Vo}\right)\,\times 0.014\times \text{N}}{\text{G}\,\left(100-\text{w}\right)}100\%$$

where:

Vx – volume of 0.01-M HCl solution used for sample titration, mL

Vo – volume of 0.01-M HCl solution used for blind sample titration, mL

0.014 – nitrogen amount corresponding to 1-mL 1-M HCl solution, g

N – molarity of used HCl solution, M×L^–1^

G – aerial dry soil weight, g

w – water percentage content in an analyzed sample.

#### Determination of Mineral Forms of Nitrogen (Ammonium and Nitrate)

Soil samples (10-g) extracted with 1% K_2_SO_4_ for 24-h were centrifuged at 2,000 rpm for 10 min, and absorbance of the supernatant [ammonium (NH_4_^+^) and nitrate (NO_3_^–^) concentrations] were measured automatically in a colorimetric nutrient flow analyzer AA100 (SEAL Analytical). In order to evaluate the NH_4_^+^ concentration, the supernatant was treated with salicylic acid, and dichloroisocyanuric acid with nitroprusside as a catalyst giving a blue solution and absorbance was determined at 660 nm (WBJ-2/IB/159). To determine the nitrate (NO_3_^–^) concentration, the supernatant was administered on a cadmium ion column. Nitrate (NO_3_^–^) in the supernatant in a phosphate buffer pH = 8.5 is reduced to nitrite (NO_2_^–^) by Cd in the ion column, and nitrite is detected by the Griess reaction method based on the conversion of sulfanilic acid (1%) to diazonium salt by reaction with NO_2_^–^ in an acid solution. The diazonium salt is then coupled to 0.1% NED (N-1-naphthylethylenediamine dihydrochloride), forming a pink azo dye that was spectrophotometrically quantified based on absorbance measurement at 540 nm.

### Genetic Fingerprint of Fungi Associated With *Trifolium repens* Rhizosphere, Roots, and Nodules

Total DNA was extracted from roots and nodules of six *T. repens* plants collected from the metal-polluted waste heap and the reference area according to Valledor et al. (2014), and from white clover rhizosphere using the MO BIO PowerSoil protocol (Qiagen). A determination of fungal genetic diversity was performed using ARISA (automated rRNA intergenic spacer analysis) fingerprinting based on an analysis of variable size of the 18S-28S rRNA internal transcribed spacer (ITS) region that, in eukaryotes, is divided into two subregions ITS1, involving 18S-5.8S rRNA genes and ITS2 comprised of 5.8S-28S rRNA genes (Ranjard et al., 2001; Johnston-Monje and Lopez Mejia, 2020). ITS1-5.8S-ITS2 region amplification was performed in a final volume of 25-μL, consisting of a 2.5-μL Fast-Start HF Reactive Buffer (0.9-mM MgCl_2_) (FastStart High Fidelity PCR System, Roche, Sigma Aldrich), 0.5-μL PCR grade nucleotide mix (100-μM of each dNTP), 0.25-μL a Fast-Start HF Enzyme Blend (1.25 U), 20.25-μL nuclease-free water, 0.5-μL DNA as a template, and 0.5-μL each of the fluorescence-labeled primers (0.04-μM), representing consensus sequences found at the 3′ end of the 18S gene for 2234C (5′-GTTTCCGTAGGTGAACCTGC-3′) and with the 5′ end of the 28S gene for 3126T (5′-ATATGCTTAAGTTCAGCGGGT-3′) (Biolegio, Netherlands). Amplification was performed in conditions as follows: initial denaturation at 94°C for 3 min, 34 cycles of denaturation at 94°C for 1 min, annealing at 55°C for 45 s, and elongation at 72°C for 1 min, and a final elongation at 72°C for 7 min. Automated electrophoresis of amplified intergenic spacer region products was performed according to the Agilent DNA 1000 assay protocol. Post-PCR products were added to the Agilent DNAchip (On-Chip Electrophoresis, Agilent Technologies, United States) for a subsequent analysis in the Agilent 2100 Bioanalyzer, equipped with DNA 2100 Expert software. The genetic diversity of fungi was determined with the R × 64 version 3.6.2 program and the StatFingerprints package (Michelland et al., 2009).

### Statistical Analysis

Results were presented as means ± SD, analyzed with one-way ANOVA, and significant differences between means were estimated with the multiple range Duncan’s test using Statistica version 13 (TIBCO).

## Results

### *Trifolium repens* Root Nodule Microsymbionts Activity

**FIGURE 1:**
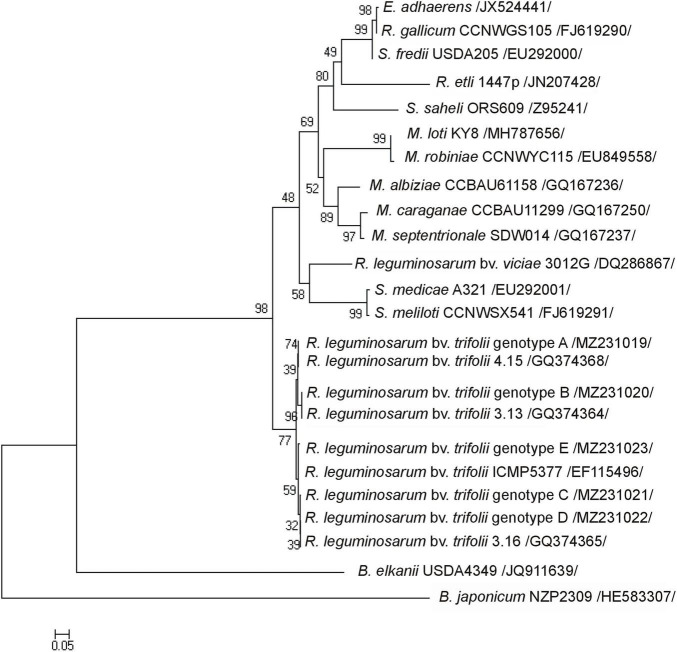
The phylogenetic Neighbor Joining tree based on 416-bp *nodA* gene sequences showing the relationship of *R. leguminosarum* bv. *trifolii* genotypes (A–E) and reference strains (the GenBank database). Numbers at nods indicate levels of bootstrap support based on an analysis of 1,000 resampled data sets. Accession numbers of reference strains representative of the studied strains are shown in parentheses. The scale bar indicates the number of substitutions per site.

*R. leguminosarum* bv. *trifolii* strains originating from the metal-polluted waste heap demonstrated a significantly higher percentage (52%) of tolerance to toxic metals in comparison to strains from the reference area (11%) (Supplementary Table 2). Plant tests showed that all studied rhizobia entered a symbiotic interaction with white clover, and as nodule inhabitants effectively transformed atmospheric nitrogen into ammonia (Table 1). Rhizobial strains of waste heap origin were similar in nitrogenase activity to rhizobia isolated from the nodules of *T. repens* growing on the reference site (Table 1). Examination of the *nodA* gene sequences, which product N-acyltransferase participates in the Nod factor formation, allowed to identify five rhizobial genotypes (A-E) (NCBI accession numbers MZ231019-23), including some specific to rhizobia, originating from the non-polluted reference area (genotypes A, D, and E), one characteristic to rhizobia from the waste heap area (genotype C), and one common to the rhizobia of waste heap and the reference area (genotype B) (Table 2). *R. leguminosarum* bv. *trifolii* reference strains (NCBI, GenBank) of *nodA* genotypes formed an independent branch in the phylogram compared to other rhizobia species (Figure 1). The *R. leguminosarum* bv. *trifolii* genotype B appeared to be the most frequent one (*f* = 0.38) among all determined genotypes; bacteria of the genotypes A, C, and D revealed frequencies of 0.17, whereas rhizobia of the genotype E showed the lowest frequency (*f* = 0.11). The 416 bp *nodA* gene sequence analysis revealed 13 variable sites involving substitutions, including nine transitions and four transversions, which influenced 138 amino acid protein sequences with 11 variable sites representing missense mutations (Table 2).

**TABLE 1:** Morphological and biochemical parameters of growth of *T. repens* inoculated with *R. leguminosarum* bv. *trifolii* from the reference area (R group), the metal-polluted waste heap (WH group), and non-inoculated with rhizobia strains (NI group).

Parameter	R group	WH group	NI group
Morphological parameters			
Leaf fresh weight	0.0051 ± 0.0011^a^	0.0045 ± 0.0010^a^	0.0018 ± 0.0005^b^
Leaf dry wet	0.0012 ± 0.0005^a^	0.0012 ± 0.0004^a^	0.0005 ± 0.0000^b^
Ash content [% f. w.]	4^a^	4^a^	2^b^
Lateral roots length [mm]	9 ± 0.56^a^	9 ± 1.12^a^	6 ± 0.95^b^
Main root length [mm]	39.22 ± 10.24^a^	40.81 ± 13.61^a^	39.48 ± 10.47^a^
Lateral roots number	2.1 ± 1.33^a^	1.75 ± 1.20^a^	1.85 ± 0.56^a^
Leaves number	3.10 ± 0.95^a^	2.95 ± 0.94^a^	2.95 ± 0.69^a^
Nodules number	4.25 ± 1.85^a^	5.8 ± 2.20^a^	0^b^
Biochemical parameters			
Chla [μg×g^–1^]	46.97 ± 4.81^a^	51.50 ± 16.15^a^	10.91 ± 3.79^b^
Chlb [μg×g^–1^]	0.045 ± 0.004^a^	0.046 ± 0.004^a^	0.08 ± 0.006^b^
Chl_a+b_[μg×g^–1^]	66.03 ± 14.69^a^	71.17 ± 12.83^a^	30.95 ± 8.88^b^
Chl_a/b_ [μg×g^–1^]	1.01 ± 0.39^a^	1.12 ± 0.32^a^	0.14 ± 0.05^b^
Proteins [mg×g^–1^]	32.89 ± 2.28^a^	67.17 ± 11.96^b^	20.25 ± 6.24^c^
Nitrogenase activity	169.65 ± 63.37^a^	163.35 ± 71.22^a^	0^b^

*Values are represented as means ± SD (n = 20). Significant differences between groups were marked with different letters.*

**TABLE 2:** Variable sites in 416-bp long fragments of the *nodA* gene (A) and corresponding amino acid sequences of NodA protein (B) of *R. leguminosarum* bv. *trifolii* strains from the metal-polluted waste heap and the reference area.

(A).															
Genotype	Number of variable site														
			
	16	97	166	178	202	217	247	253	272	283	340	394	402	Frequency	Strains
A	T	C	T	T	T	T	C	C	G	C	G	G	C	0.17	1.6 K, 1.7 K, 4.3 K, 4.4 K, 4.8 K, 4.10 K, 5.5 K
B	C	.	.	C	.	.	A	G	.	.	A	T	A	0.38	2.9 K, 9.9 K, 4.51 H, 5.1 H, 5.2 H, 5.5 H, 6.3 H, 6.5 H, 6.12 H, 6.13 H, 7.1 H, 7.2 H, 7.3 H, 7.4 H, 7.6 H, 7.7 H
C	.	A	C	T	C	C	C	C	A	T	G	.	.	0.17	3.3 H, 3.5 H, 4.1 H, 4.2 H, 4.3 H, 4.4 H, 4.5 H
D	T	.	.	.	.	.	.	.	.	.	.	.	.	0.17	3.2 K, 5.3 K, 5.7 K, 5.10 K, 6.5 K, 6.6 K, 8.8 K
E	.	.	T	.	.	.	.	.	.	.	.	.	.	0.11	3.3 K, 3.5 K, 3.9 K, 9.2 K, 9.3 K


(B).															
Genotype	Number of variable site														
	6	33	56	60	73	83	85	91	95	114	131
A	F	H	C	Y	F	L	P	R	H	V	A
B	L	.	.	H	.	I	A	.	.	M	S
C	.	N	R	Y	L	L	P	Q	Y	.	.
D	F	.	.	.	.	.	.	.	.	.	.
E	.	.	C	.	.	.	.	.	.	.	.

*A, alanine; C, cysteine; F, phenylalanine; H, histidine; I, isoleucine; L, leucine; M, methionine; N, aspartic acid; P, proline; Q, glutamic acid; R, arginine; S, serine; Y, tyrosine; V, valine.*

The *in vitro* tests indicated that all studied *R. leguminosarum* bv. *trifolii* strains showed potential to promote plant growth (Supplementary Table 3); 32% of the strains appeared positive for all six traits tested, 16% were positive for five traits, 37% of the tested strains were positive for four traits, and 21% for three traits. About 95% of the studied rhizobial strains showed the ability to synthesize indole-3-acetic acid, 90% produced ACCD, 84% were able to solubilize phosphates, 63% were active producers of organic acids and siderophores, while 47% synthesized acetoin (Supplementary Table 3). The *in vitro* tests revealed significant differences in potential PGP traits between rhizobial strains of waste heap and the reference area origin. The rhizobial strains from the metal-polluted area were significantly more effective (80%) in acetoin production than strains from the reference area (11%), whereas more strains from the reference area (100%) could synthesize IAA than rhizobial strains from the Bolesław waste heap (80%) (Table 3). Quantitative analysis revealed similar concentrations of IAA and ACCD for *R. leguminosarum* bv. *trifolii* strains originating from the metal-polluted waste heap and the non-polluted reference area (Table 3). There were significant differences in the numbers of strains with regard to positive or negative reactions in the *in vitro* tests. Almost 50% of the *R. leguminosarum* bv. *trifolii* strains originating from the metal-polluted area appeared positive for all six tested traits, 30% were positive for four tested characteristics, and 10% were positive for five and three tested traits, while, among the rhizobia from the reference area, 11% were positive for all six as well five studied traits, 44 and 34% of the strains appeared positive, respectively, for four and three evaluated characteristics (Supplementary Table 3).

**TABLE 3:** Potential plant-growth-promoting traits of *R. leguminosarum* bv. *trifolii* strains isolated from nodules of *T. repens* from the metal-polluted waste heap (WH) and non-polluted reference grassland (R).

Plant growth promotion trait	WH	R
Acetoin [%]	80	11*
ACCD [%]	90	89
Mean ACCD activity [μM α-ketobutyrate×mg protein^–1^]	0.064 ± 0.09	0.062 ± 0.16
IAA [%]	80	100
Mean IAA concentration [μg×mL^–1^]	50.17 ± 14.26	35.79 ± 18.71
Organic acids [%]	70	56
Siderophores [%]	70	56
P solubilization [%]	90	78

*Significant differences between groups were marked with an asterisk.*

### Effects of *Rhizobium leguminosarum* bv. *trifolii* on Growth of White Clover

#### Morphological and Biochemical Parameters

**FIGURE 2:**
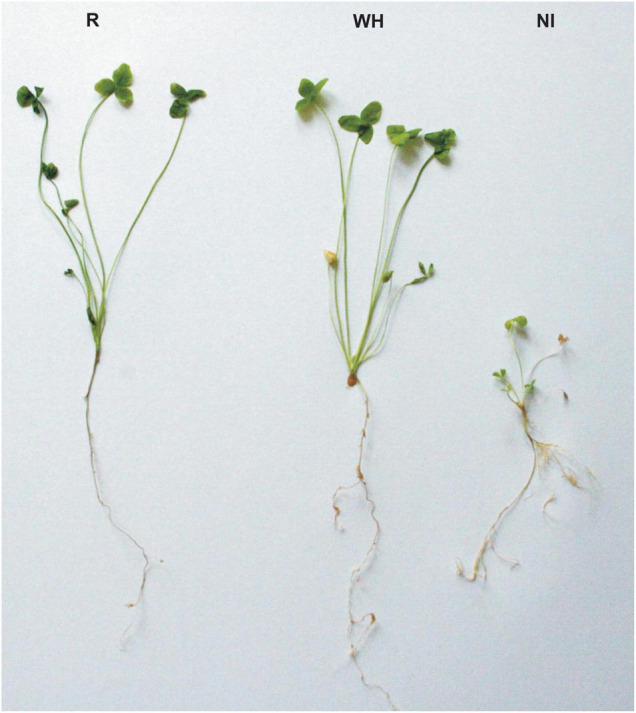
The growth habit of *Trifolium repens* inoculated with *R. leguminosarum* bv. *trifolii* from the non-polluted reference Bolestraszyce area (R), the Bolesław metal-polluted waste heap area (WH), and not inoculated with rhizobia (NI).

The fresh and dry weight, ash content, total root length, and mean length of lateral roots as well as mean numbers of nodules of *T. repens* inoculated with *R. leguminosarum* bv. *trifolii* strains from both metal-polluted and non-polluted origin were significantly higher in comparison to non-inoculated plants (Table 1). No significant differences were observed for these plant-growth parameters between *T. repens* inoculated with rhizobia from the metal-polluted waste heap and from the reference area (Figure 2). The concentrations of photosynthetic pigments in leaves of white clover plants inoculated with rhizobial strains from the metal-polluted area and the reference area were similar, whereas the protein concentration was significantly higher in *T. repens* inoculated with rhizobia from the metal-polluted waste heap than in plants inoculated with rhizobia from the non-polluted reference area (Table 1).

#### Anatomical Analysis of *Trifolium repens* Root Nodule

##### Light Microscopy Examination

**FIGURE 3:**
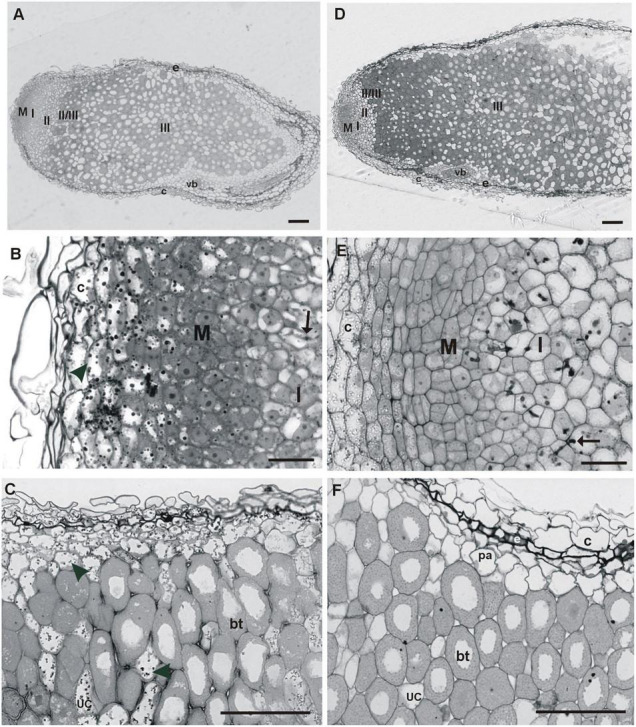
Longitudinal sections of fully developed *T. repens* root nodules from the metal-polluted waste heap **(A–C)** and non-polluted reference **(D–F)** area. M—meristem, I—infection thread penetration zone, II—early symbiosis zone, II/III—inter-zone with large amyloplasts, III—nitrogen fixation zone; arrow—infection thread, arrowhead—precipitates in vacuoles, bt—bacteroidal tissue, c—nodule cortex, e—nodule endodermis, pa—nodule parenchyma, uc—uninfected cell, vb—vascular bundle. Scale bars on images **(A,B)** correspond to 100 μm, while scale bars on images **(C–F)** correspond to 50 μm.

The root nodules of *T. repens* growing on the metal-polluted waste heap in Bolesław and the non-polluted reference area are of the indeterminate type and, by consequence, characterized by typical zonation (Figures 3A,D), i.e., zone of: meristem, infection thread penetration, early symbiosis, nitrogen fixation, and the senescence one. Nodules of waste heap and non-polluted reference origin plants were surrounded by peripheral tissues, which consisted of a nodule cortex, nodule endodermis, and nodule parenchyma (Figures 3A,D). The outer cortex and parenchyma of the nodules from the waste heap plants consisted of large, thick-walled cells, with vacuoles filled with dark precipitates (Figure 3C). In nodule tissues of plants from the reference area, such precipitates were not detected (Figure 3F). The outer cortex of both, waste heap and reference origin plant nodules, was separated from the parenchyma by a dark-stained layer of endodermis (Figures 3C,F). The nodules were composed of an apical persistent meristem, vascular system, nodule cortex, and the central tissue, containing nitrogen-fixing forms of rhizobia (bacteroids) (Figures 3A,D). The apically situated meristem consisted of small dividing cells, and, unlike the reference plant nodules, in the waste heap origin ones both, meristematic cells and adjacent cortex cells, contain large dark precipitates in vacuoles (Figures 3B,E). Below the meristem of waste heap origin nodules, the infection thread penetration zone (or the early symbiosis zone, zone I) was penetrated by a few, small infection threads (Figure 3B) in contrast to the reference origin nodules (Figure 3E), in which the infection thread penetration zone comprised of many large, long infection threads. Beneath, the infection thread penetration zone, infected cells containing large amyloplasts characteristic for an inter-zone II/III (Figures 3A,D), were observed. Next, the nitrogen-fixing zone (III) of bacteroidal tissue, infected cells with dense cytoplasm, numerous symbiosomes, and small amyloplasts situated close to the intercellular spaces were detected. In the waste heap origin plant nodules, the zone III cells were separated by non-infected cells with vacuoles filled with dark precipitates, whereas, in corresponding cells of the reference area origin nodules, such dark material in vacuoles was not detected (Figures 3A,D).

##### Transmission Electron Microscopy Nodule Investigation

**FIGURE 4:**
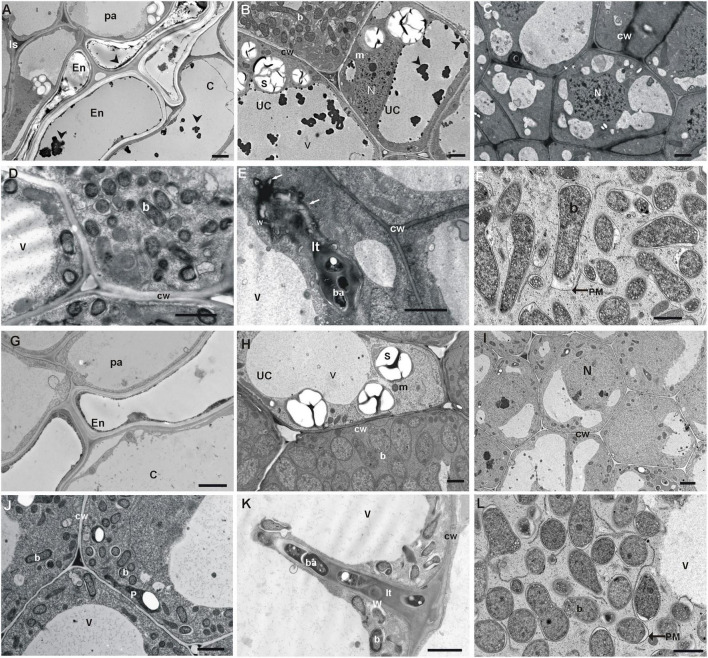
TEM micrographs of waste heap origin *T. repens* nodules **(A–F)** and non-polluted reference ones **(G–L)**. **(A)** Nodule cortex tissues: thick-walled parenchyma (pa) cells with dark inclusion in intercellular spaces (Is), and double-layer nodule endodermis (En), and cortex cells with dark precipitates (an arrowhead) in their vacuoles. **(B)** Visible dark inclusions (an arrowhead) in central and small vacuoles of uninfected cells (uc). **(C,D)** Abnormal thick-walled cells in the thread penetration zone and the early symbiosis zone. **(E)** Abnormal thick-walled infection thread with lateral bulges (arrows) containing electron-dense depositions of matrix material. **(F)** Infected cells from the early symbiosis zone and symptoms of early bacteroids degradation (peribacteroidal membrane—PM outgrowths and widening of the peribacteroidal space (an asterisk). **(G–L)** Control nodules and typical nodule cortex tissues with thin-walled parenchyma (pa) cells; one layer of endodermis and cortex cells without inclusion in vacuoles **(G)**. **(H)** Typical vacuoles of bacteroidal tissue devoid of precipitates. **(I,J)** Typical thin-walled cells of bacteroidal tissues. **(K)** Thin-walled infection threads and bacteria endocytosis from an un-walled thread tip. **(L)** Typical bacteroid differentiation and early symptoms of bacteroids degradation. b, bacteroid; ba, bacterium; cw, cell wall; It, infection thread; m, mitochondrion; N, nucleus; p, plastid; s, starch granule; v, vacuole; w, infection thread wall. Scale bars on images correspond to 2 μm.

TEM analysis revealed that the three-layer cortex cells of white clover nodules were strongly vacuolated, possessed few organelles, and these originating from nodules of the Bolesław waste heap area contained vacuolar dark precipitates (Figures 4A,G). The nodule endodermis of reference origin plant nodules was single-layered and consisted of flat cells devoid of precipitates (Figure 4G), whereas the endodermis of waste heap origin plant nodules was double layered with vacuolar dark precipitates (Figure 4A). The waste heap origin nodule parenchyma, as well as endodermis cell walls, were substantially thicker than in the reference area origin nodules. Moreover, the endodermis of waste heap origin nodules consisted of characteristically striated suberized layers (Figures 4A,G).

The apically situated meristem of the white clover nodules from both, waste heap as well as reference area origin, consisted of poorly vacuolated small dividing cells surrounded by thick walls. Vacuoles of waste heap origin plant nodules contained numerous dark precipitates (Figure 4B). In contrast, the reference origin nodules did not contain such vacuolar inclusions (Figure 4H). Only in *T. repens* nodules originating from the waste heap, some vacuoles of infected cells were also filled with granular material that sometimes replaced the central vacuole.

In the nodules of waste heap clover plants, the walls of cells of the infection threads penetration zone were substantially thicker (Figure 4C) than these of reference origin ones (Figure 4I), and consisted of many layers of fibrous material with numerous membrane invaginations that were not observed in nodules of plants from the non-polluted reference area. Thick walls of infection threads can cause disturbances in the bacteria release and endocytosis that was found in fully developed infected cells of waste heap origin nodules (Figure 4E) but not in non-polluted reference origin ones (Figure 4I). However, no abnormalities in the formation of bacteroids were observed in *T. repens* nodules regardless of their waste heap or reference area origin. In nodules of both origins, the process of bacteroid degradation began early in the zone of young symbiosis, where deformations of peribacteroidal membranes and widening of peribacteroidal spaces of young bacteroids were observed (Figure 4).

**FIGURE 5:**
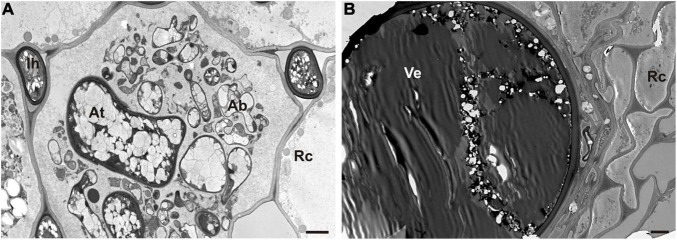
TEM micrographs of symbiotic mycorrhizal fungi in roots of nodulated *T. repens* from the metal-polluted waste heap area. **(A)** Arbuscule (Ab) and intercellular hyphae (Ih) of arbuscular mycorrhiza fungi in root cortex cells (Rc). At, arbuscule trunk. **(B)** Visible large vesicle (Ve) inside root cortex cells (Rc). Scale bars correspond to 2 μm.

The microscopic analysis also showed the occurrence of AMF in the roots of waste heap plants (Figure 5), in contrast to plants originating from the reference area where no mycorrhiza was found. Hyphae, arbuscules, and vesicles of the AMF were found in the parenchyma cells of the root cortex (Figure 5).

### Plant Types of Toxic Metals Adaptation (Accumulator vs. Excluder) and Nutrient Soil Resources

Significantly higher concentrations of Zn, Pb, and Cd were found in the soil of the Bolesław waste heap in comparison to the non-polluted reference one. Moreover, metal concentrations in leaves and roots of *T. repens* from the metal-polluted area were significantly higher compared to those in leaves and roots of plants from the non-polluted area. Leaves of *T. repens* contained substantial concentrations of metals, but these were significantly lower than in the roots showing thus clearly an excluder strategy (Table 4). The concentrations of Zn were higher than those of Pb and Cd.

**TABLE 4:** Zinc, lead, and cadmium concentrations (mg×kg^–1^ dry soil) as well in roots and leaves of *T. repens* originating from the metal-polluted waste heap (WH) and the non-polluted reference (R) area.

	Soil			Root			Leaves		
Study area	Zn	Pb	Cd	Zn	Pb	Cd	Zn	Pb	Cd
WH	50,008 ± 9,356^a^	5,008 ± 998^a^	490 ± 26.89^a^	2,390 ± 553^a#^	2,310 ± 118^a#^	156 ± 12.14^a#^	289 ± 56.47^a##^	90.18 ± 15.89^a##^	2.46 ± 0.45^a##^
R	83.41 ± 11.39^b^	9.86 ± 0.87^b^	2.51 ± 0.21^b^	52.46 ± 10.59^b#^	2.88 ± 0.27^b#^	0.26 ± 0.09^b#^	39.36 ± 9.25^b##^	0.52 ± 0.13^b##^	0.01 ± 0.004^b##^

*Values were presented as means ± SD. Significant differences between values derived from the waste heap and reference areas were marked with different letters in a superscript. Significant differences between values received from the plant organs were marked with a number sign.*

The soil originating from the waste heap area had a lower dry weight as well as lower concentrations of ammonium and nitrate compared with the reference area (Table 5). No significant differences in total Kjeldahl nitrogen were found between the two soils (Table 5). The soil from the waste heap contained higher concentrations of calcium and magnesium and lower concentrations of potassium and sodium than the reference grassland (Table 5).

**TABLE 5:** Macroelements concentration in the metal-polluted waste heap (WH) and non-polluted reference (R) soils.

Concentration (in soil d.w.)	WH	R
Ca [mg×kg^–1^]	14,250 ± 8,586	3,179 ± 596*
K [mg×kg^–1^]	7,312 ± 2,059	22,489 ± 1,064*
Na [mg×kg^–1^]	570 ± 117	6,327 ± 741*
Mg [mg×kg^–1^]	7,739 ± 561	2,403 ± 465*
N_og_ [%]	0.23 ± 0.10	0.12 ± 0.05
N-NH_4_^+^ [mg×kg^–1^]	3.36 ± 0.57	5.04 ± 2.25*
N-NO_3_^–^ [mg×kg^–1^]	0.38 ± 0.13	3.11 ± 0.53*
d.w. [%]	77.70 ± 2.77	88.93 ± 3.49*

*d.w., dry weight. Significant differences between groups were marked with an asterisk.*

### Mycorrhizal Fungi Associated With *Trifolium repens* Roots

**FIGURE 6:**
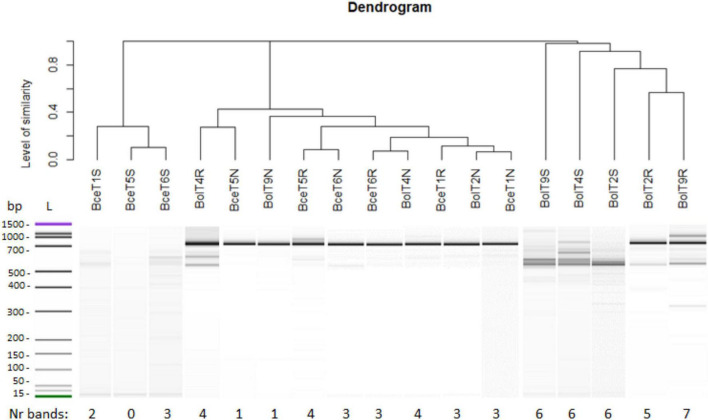
Genetic diversity of arbuscular mycorrhizal fungi (AMF) isolated from samples of soil (S), roots (R), and nodules (N) of *T. repens* from the metal-polluted waste heap (Bol) and the non-polluted reference area (Bce).

The ITS1-5.8S-ITS2 rRNA gene region length analysis revealed a low genetic diversity of mycorrhizal fungi in the rhizosphere of *T. repens* growing on the non-polluted reference area, and a richer genetic diversity (more bands, amplicons) of fungal symbionts in the rhizosphere of white clover from the metal-polluted waste heap (Figure 6). The roots of *T. repens* from the metal-polluted soil showed also more diverse fingerprint patterns of fungal symbionts than the root of plants from the non-polluted area. Clear dominance of one fungus fingerprint amplicon in the nodules of both polluted and non-polluted areas, clustering together in the dendrogram, was found (Figure 6).

## Discussion

The results of this study demonstrate that *R. leguminosarum* bv. *trifolii* bacteria isolated from nodules of *T. repens* growing on the polluted Bolesław waste heap harbor traits that can be beneficial to plant growth and health. Rhizobia, together with their host plant, which is a metal excluder, enter into a symbiosis with AMF, which have (AMF means arbuscular mycorrhizal fungi, fungi is a plural form) the potential to be adopted in phytostabilization. Chaudri et al. (1993) and Giller et al. (1998) reported that, on metal-polluted areas (Woburn, United Kingdom) *R. leguminosarum* bv. *trifolii* bacteria formed an ineffective symbiosis with clover, and were classified into only a few genotypes. In this study, we show that rhizobia from an old metal-polluted waste heap are tolerant to metals, enter into symbiosis with white clover, exhibit substantial nitrogenase activity, and are equipped with nodulation genes (i.e., *nodA*). The *nodA* gene is a member of the canonical core *nodABC* genes and encodes an enzyme that is involved in the transfer of an N-acyl residue into a lipochitooligosaccharide Nod factor, which plays a crucial role in nodule development. The Nod molecule is a host-specificity determinant that triggers, *i.a.*, the plant cell developmental program resulting in the formation of a nodule, the entry of rhizobia into plant cells, and the progress in nodule formation (Franche et al., 2009). Moreover, we found that rhizobia from the waste heap area do not differ in nodulation ability as well as nitrogenase activity from rhizobia from the non-polluted area (Table 1 and Supplementary Table 1). Consequently, metal-tolerant rhizobia from the metal-polluted area can be considered as effective microsymbionts of leguminous plants on the extremely high-metal-polluted Bolesław waste heap.

Bacteria-induced improvement of plant fitness on metal-polluted soils may be achieved by direct interactions of microorganisms with metals, i.e., modifications of the cellular barrier permeability, preventing the transfer of metal ions into the cytoplasm, efflux of metal ions out of the cell, enzymatic reduction of metal ions, extracellular sequestration of metal ions by bacterial metabolites, and intracellular sequestration of ions (Ji and Silver, 1995; Silver and Phung, 1996; Bruins et al., 2000; Oleńska and Małek, 2013a) or/and by indirect beneficial effects on plant growth on metal-polluted sites (Oleńska et al., 2020a). It was demonstrated that *R. leguminosarum* bv. *trifolii* bacteria from the metal-polluted waste heap are equipped with some adaptation mechanisms to metals. For instance, they possess a gene, whose product may be potentially involved in chemiosmotic removal of Cd (Oleńska and Małek, 2013b). Moreover, it has also been shown recently that the *R. leguminosarum* bv. *trifolii* bacteria from the waste heap produce exopolysaccharides that differ substantially from the exopolysaccharides of rhizobia from the reference soil in both qualitative and quantitative compositions, suggesting a significant role of these compounds in direct interactions of rhizobia with metals (Oleńska et al., 2021). In biofilm studies, rhizobia from the waste heap show a significantly higher survival rate in non-exposed as well as metal-exposed conditions in comparison with strains from the non-polluted area, implying the adaptation of the waste heap strains to metals (Oleńska et al., 2021).

The present study shows that *R. leguminosarum* bv. *trifolii* bacteria from the metal-polluted waste heap possess traits that have the potential to indirectly improve the tolerance of their host to toxic metals through a beneficial influence on plant growth (Table 3). The percentage of rhizobia isolated from nodules of plants growing on the metal-polluted waste heap showing acetoin production is higher than for those from the non-polluted area. Acetoin (3-hydroxy-2-butanone) is considered as a significant inducer of induced systemic resistance (ISR) (Wang et al., 2009) and as a volatile organic compound (VOC), playing a role in the bacterial life cycle (e.g., regulation of bacterial motility, antibiotic resistance, biofilm formation) and as a compound participating in bacterial association with host plants (e.g., increases of plant biomass, fruit yield, seed production, lateral root and root hair formation, nutrient uptake, and photosynthetic activity) (Sharifi and Ryu, 2018a,b; Morcillo et al., 2020a,b). Oleńska et al. (2020b) reported that in *in vitro* studies also other bacterial taxa exhibit traits that can promote plant growth in conditions of toxic metal concentrations. For example, *Bacillus thuringiensis*, *Chryseobacterium lathyri*, *Pseudomonas putida*, *Bacillus cereus*, *Stenotrophomonas maltophilia*, which are also inhabitants of nodules of *T. repens* growing on the Bolesław waste heap, showed able to, e.g., synthesize acetoin, siderophores, IAA, ACCD, fix N_2_, solubilize phosphates, as well as to tolerate exposure to increased metal concentrations. Furthermore, Sánchez-López et al. (2018) revealed that, in case of high concentrations of Zn/Pb/Cd in soil, the *Methylobacterium* sp. strain Cp3 possesses potentially beneficial traits, i.e., plant growth promotion and metal tolerance. It is also known that bacteria producing siderophores, which are low molecular (400–1,500 Da) weight chelators, with a high affinity for unavailable Fe(III) and transforming it into Fe(II), supply plants with iron that is crucial for chlorophyll synthesis, maintenance of chloroplast structure and function, DNA synthesis, respiration, and is a constituent of many enzyme prosthetic groups (Rout and Sahoo, 2015; Jian et al., 2019). Shi et al. (2017) reported enhanced siderophore synthesis in *Pseudomonas aeruginosa* under Cd(II) and Zn(II) stress, while Złoch et al. (2016) showed synthesis of hydroxamate-, catecholate-, and phenolate-type siderophores production by *Streptomyces* sp. from *Betula pendula* and *Alnus glutinosa* growing in the presence of Cd(II). The *Bacillus* spp. strain PZ-1 synthesized hydroxamate-type siderophores when exposed to toxic Pb(II) concentrations and enhanced the storage of this metal in the underground tissues of *Brassica juncea* (Yu et al., 2017; Jinal et al., 2019). Auxins are key regulators of plant development, e.g., cell division, expansion, and differentiation (Paque and Weijers, 2016). Increased expression of IAA was reported, for instance, for *Pseudomonas grimontii* strain Bc09, *Pantoea vagans* strain So23, *Pseudomonas veronii* strain E03, and *Pseudomonas fluorescens* strain Oj24 that positively influenced biomass production of switchgrass under Cd(II) stress (Begum et al., 2019). Similarly, *Leifsonia xyli* strain SE134 under Cu exposure showed an enhanced IAA synthesis (Kang et al., 2017). In plants inoculated with ACCD-producing bacteria, longer roots and higher resistance to pathogens were reported (Ravanbakhsh et al., 2017; Ghosh et al., 2018; Saikia et al., 2018; Gupta and Pandey, 2019). Han et al. (2015) showed a positive effect of ACCD-producing *Pseudomonas stutzeri* A1501 strain on rice biomass. Solubilization of inorganic phosphates by bacteria is predominantly performed as the result of production of organic acids (Zhao and Zhang, 2015; Naraian and Kumari, 2017). Oves et al. (2017) revealed that, under metal stress, *Ensifer adhaerens* strain OS3 is a phosphate solubilizer and a chromium reducer. Taking into consideration that the rhizobia from the metal-polluted waste heap exhibit positive traits *in vitro*, we may assume that these bacteria may accomplish a substantial role in improving plant growth, including these able to accumulate toxic metals and being useful for remediation purposes.

Rhizobia from both, the waste heap and the reference area, influence positively the growth of *T. repens* (Table 1 and Figure 2). No significant differences in chlorophyll *a* and *b*, the sum of chlorophylls, the chlorophyll *a* to *b* ratio, except protein concentration, were found between white clover inoculated with rhizobia from the waste heap and the reference area. Therefore, it can be assumed that *R. leguminosarum* bv. *trifolii* bacteria originating from the waste heap can be proposed as endophytes efficient to improve the growth of *T. repens* in conditions of high metal pollution, where phytostabilization is required. Bidar et al. (2007) suggested that *T. repens* can be used as a phytostabilizing plant species in metal-polluted areas; Oleńska et al. (2020b) suggested that white clover has the potential to be used for phytostabilization on the Bolesław waste heap. The results of the present study demonstrate that *T. repens* accumulates metals in roots and leaves, and that the root is the predominant location of metal accumulation (Table 4). Light and transmission electron microscopy investigation of nodules of *T. repens* growing on the waste heap revealed morphological adaptations of plants to toxic metals that are manifested predominantly as apoplast modifications (Figures 3, 4). Suberinization of the cortex cell walls, as well as the presence of granules in their vacuoles, suggest redirection of stored material (conceivably polyphenols and metals connected with organic acids) into the apoplast, what was found exclusively in plants from the waste heap and can be considered as adaptations to metal stress. Redirection of ions in extracellular (i.e., cell walls) and intracellular (i.e., vacuole) spaces is the first line of plant defense against toxic metals (Hasan et al., 2017). After entering into root cells, metal ions can form complexes with different ligands, e.g., organic acids and as carbonate, sulfate, or phosphate precipitates, which are accumulated among others in vacuoles, preventing the accumulation of free metal ions in a cytosol (Ali et al., 2013; Yan et al., 2020).

Ważny et al. (2021) claimed that mycorrhiza may serve as a significant, sufficient constituent of plant adaptations to metal-polluted areas. Mycorrhizal fungi can enhance the metal tolerance of their host plants by increasing the uptake of water, nutrients, as well as plant growth-promoting traits, e.g., by binding metals in the mycelium (Luo et al., 2014). In mycorrhized plants under metal stress, Turnau (1998) reported preferential accumulation of metals in intraradical fungal structures rather than in root tissues. Yang et al. (2016) reported a higher Pb uptake in *Robinia pseudoacacia* roots mycorrhized with *Rhizophagus intraradices* compared to non-mycorrhizal plants. Also, biomass as well as nutrients (i.e., N, P, S, and Mg) uptake were reported to increase in case of AMF presence in roots. In addition, these authors found that the presence of other mycorrhized legume herbs, i.e., *Trifolium pratense* and *Medicago sativa* increased the mycorrhization of *R. pseudoacacia*, possibly due to a common signaling pathway turned on by mycorrhiza and rhizobia. Leguminous plants also lowered soil pH, resulting in higher availability of Pb ions. Dhalaria et al. (2020) reported a substantial role of mycorrhizal fungi vesicles in the accumulation of metals as well as the synthesis of glycoprotein chelators (e.g., glomalin) by mycorrhizal fungi and enhancing the antioxidative responses in metal stress conditions. Our microscopic analyses of *T. repens* roots revealed the presence of AMF only in plants from the metal-polluted waste heap (Figure 5). This may suggest that these AMF fulfill an important role in the adaptation of the plants to the extreme conditions on the waste heap. Martínez-Hildago and Hirsch (2017) reported a substantial role for AMF in the selection of bacterial nodule residents, and Rodríguez-Caballero et al. (2017) showed changes in the plant bacterial communities under influence of AMF. It was also demonstrated that AMF may trigger plant metabolism, e.g., induce a systemic response through leaf protein expression (Lingua et al., 2012). Moreover, Scheublin et al. (2004) reported arbuscular mycorrhizal fungi invasion of legume nodules, and that AMF communities vary depending on plant species as well as parts of a root system.

## Concluding Remarks and Future Prospects

The present study shows that the Bolesław waste heap, besides the occurrence of very high metal concentrations, is also deficient in nutrients, including bioavailable forms of nitrogen, i.e., ammonium and nitrate ions (Table 5). It is obvious that the presence of rhizobia, effectively fixing nitrogen and reducing it into ammonia that is available to leguminous plants, is of significant importance for their hosts, inhabiting such challenging metal-polluted and nutrient-poor areas. Also, the presence of genetically diverse AMF communities in roots and nodules of *T. repens*, originating from the metal-polluted waste heap area, is promising in the function of protection of leguminous plants against toxic metals. However, the taxonomy and characteristics of these AMF still must be studied more in detail. Taking into consideration that leguminous plants, entering in symbiotic interaction with rhizobia fixing N_2_, are pioneers on nutrient-poor soils (Padilla and Pugnaire, 2006; Li et al., 2007; Fterich et al., 2014; Roa-Fuentes et al., 2015), we may conclude that *R. leguminosarum* bv. *trifolii*, mycorrhizal fungi, and *T. repens*, together constituting a metaorganism (Thijs et al., 2016), can be a promising tool for phytostabilization of Zn-, Pb-, and Cd-polluted soils.

## Data Availability Statement

The data presented in the study are deposited in the NCBI GenBank repository, accession numbers MZ231019-23.

## Author Contributions

EO contributed to conceptualization, data curation, project administration, resources, and writing the original draft. EO, MS-R, ST, SS, and OA contributed to formal analysis. EO and JV contributed to funding acquisition. EO and ST investigated the study and contributed to software. TW, ST, SS, EO, and MS-R contributed to methodology. JV and WM contributed to supervision. MW, IS, and WM contributed to validation. EO, ST, and MS-R contributed to visualization. JV, MW, WM, and MS-R contributed to writing, reviewing, and editing. All authors contributed to the article and approved the submitted version.

## Conflict of Interest

The authors declare that the research was conducted in the absence of any commercial or financial relationships that could be construed as a potential conflict of interest.

## Publisher’s Note

All claims expressed in this article are solely those of the authors and do not necessarily represent those of their affiliated organizations, or those of the publisher, the editors and the reviewers. Any product that may be evaluated in this article, or claim that may be made by its manufacturer, is not guaranteed or endorsed by the publisher.
