# Supplementary material for: An Alliance of Trifolium repens—Rhizobium leguminosarum bv. trifolii—Mycorrhizal Fungi From an Old Zn-Pb-Cd Rich Waste Heap as a Promising Tripartite System for Phytostabilization of Metal Polluted Soils
Source: Front Microbiol. 2022 Apr 15;13:853407. doi: 10.3389/fmicb.2022.853407 (PMC9051510; doi:10.3389/fmicb.2022.853407)
Supplement: Supplementary file 1 [file Data_Sheet_1.docx]

Supplementary Material

Table S1. Analytical characteristics of the ICP-OES and spectrophotometric methods used for quantification of toxic metals, nutrients, IAA, and α-ketobutyrate.

| **standard** | Calibration curve equation  y=(a±SD)x+(b±SD), n=3 |
| --- | --- |
| Zn | y=(0.0004095156±)x-(0.0057629±) |
| Pb | y=(0.0062335±)x+(0.003071146±) |
| Cd | y=(0.0005024621±)x-(0.000670±) |
| Ca | y=(0.009404±)x-(0.0716657±) |
| Mg | y=(0.0010405±)x+(0.0053369±) |
| K | y=(0.001496±)x+(0.0022509±) |
| Na | y=(0.0001401824±)x-(0.1828504±) |
| IAA | y=(0.00027±)x-(0.0041±) |
| α-ketobutyrate | y=(1.4673±)x+(0.03267±) |

Table S2. Zinc (Zn), lead (Pb), and cadmium (Cd) tolerance, nodulation ability, and nitrogenase activity of *R.* *leguminosarum* bv. *trifolii* strains isolated from nodules of *T. repens* growing on the metal polluted waste heap (H) and the non-polluted reference (K) area presented in a binary system (0, negative, 1, positive).

| strain | Zn | | Pb | | Cd | | nodulation ability | nitrogenase activity  [nM ethylene h^-1^ plant^-1^] |
| --- | --- | --- | --- | --- | --- | --- | --- | --- |
|  | 0.1 mM | 2.5 mM | 0.1 mM | 2.5 mM | 0.1 mM | 1 mM |  |  |
| 1.6K | 1 | 1 | 1 | 0 | 1 | 0 | 1 | 233.12 |
| 1.7K | 1 | 1 | 1 | 1 | 1 | 0 | 1 | 256.66 |
| 2.9K | 1 | 1 | 1 | 1 | 1 | 0 | 1 | 189.89 |
| 3.2K | 1 | 1 | 1 | 1 | 1 | 0 | 1 | 89.58 |
| 3.3K | 1 | 1 | 1 | 1 | 1 | 0 | 1 | 115.13 |
| 3.5K | 1 | 1 | 1 | 1 | 1 | 0 | 1 | 189.54 |
| 3.9K | 1 | 1 | 1 | 1 | 1 | 1 | 1 | 257.28 |
| 4.3K | 1 | 1 | 1 | 1 | 1 | 0 | 1 | 57.35 |
| 4.4K | 1 | 1 | 1 | 1 | 1 | 0 | 1 | 249.12 |
| 4.8K | 1 | 1 | 1 | 1 | 1 | 0 | 1 | 45.56 |
| 4.10K | 1 | 0 | 1 | 1 | 1 | 0 | 1 | 139.46 |
| 5.3K | 1 | 1 | 1 | 1 | 1 | 0 | 1 | 212.44 |
| 5.5K | 1 | 0 | 1 | 1 | 1 | 1 | 1 | 268.32 |
| 5.7K | 1 | 1 | 1 | 1 | 1 | 0 | 1 | 185.76 |
| 5.10K | 1 | 1 | 1 | 1 | 1 | 0 | 1 | 170.20 |
| 6.5K | 1 | 1 | 1 | 1 | 1 | 0 | 1 | 212.45 |
| 6.6K | 1 | 1 | 1 | 1 | 1 | 1 | 1 | 95.37 |
| 8.8K | 1 | 1 | 1 | 1 | 1 | 0 | 1 | 129.57 |
| 9.2K | 1 | 1 | 1 | 1 | 1 | 0 | 1 | 168.21 |
| 9.3K | 1 | 1 | 1 | 1 | 1 | 0 | 1 | 208.12 |
| 9.9K | 1 | 1 | 1 | 1 | 1 | 0 | 1 | 89.63 |
| 3.3H | 1 | 1 | 1 | 1 | 1 | 1 | 1 | 258.57 |
| 3.5H | 1 | 1 | 1 | 1 | 1 | 0 | 1 | 123.71 |
| 4.1H | 1 | 1 | 1 | 1 | 1 | 1 | 1 | 132.36 |
| 4.2H | 1 | 1 | 1 | 1 | 1 | 1 | 1 | 220.53 |
| 4.3H | 1 | 1 | 1 | 1 | 1 | 0 | 1 | 258.57 |
| 4.4H | 1 | 1 | 1 | 1 | 1 | 0 | 1 | 123.71 |
| 4.5H | 1 | 0 | 1 | 0 | 1 | 0 | 1 | 109.29 |
| 4.51H | 1 | 1 | 1 | 1 | 1 | 0 | 1 | 224.78 |
| 5.1H | 1 | 1 | 1 | 1 | 1 | 0 | 1 | 60.96 |
| 5.2H | 1 | 1 | 1 | 1 | 1 | 0 | 1 | 41.25 |
| 5.5H | 1 | 1 | 1 | 1 | 1 | 0 | 1 | 274.10 |
| 6.3H | 1 | 1 | 1 | 1 | 1 | 1 | 1 | 78.65 |
| 6.5H | 1 | 1 | 1 | 1 | 1 | 1 | 1 | 142.38 |
| 6.12H | 1 | 1 | 1 | 1 | 1 | 1 | 1 | 218.45 |
| 6.13H | 1 | 1 | 1 | 1 | 1 | 1 | 1 | 248.23 |
| 7.1H | 1 | 1 | 1 | 1 | 1 | 1 | 1 | 135.85 |
| 7.2H | 1 | 1 | 1 | 1 | 1 | 1 | 1 | 118.18 |
| 7.3H | 1 | 1 | 1 | 1 | 1 | 1 | 1 | 218.57 |
| 7.4H | 1 | 1 | 1 | 1 | 1 | 0 | 1 | 81.59 |
| 7.6H | 1 | 1 | 1 | 1 | 1 | 0 | 1 | 162.48 |
| 7.7H | 1 | 1 | 1 | 1 | 1 | 1 | 1 | 198.18 |

Table S3. Potential plant-growth promoting traits of *R. leguminosarum* bv. *trifolii* strains isolated from nodules of *T. repens* originating from the metal polluted waste heap and the non-polluted reference area. Abbreviations: IAA – indole-3-acetic acid synthesis, ACCD – deaminase ACC activity, sid. – siderophores production, org acids – organic acids production, acet. – acetoin production, P solub. – phosphate solubilization, IS – index of solubilization.

| No. | Bacterial strain | **Plant-growth promotion traits** | | | | | | | |
| --- | --- | --- | --- | --- | --- | --- | --- | --- | --- |
|  |  | IAA | IAA conc. | ACCD | ACCD activity | sid. | org acids | acet. | P solub.  IS |
| 1 | 1.6K | 1 | 35.19 | 1 | 0.68 | 1 | 1 | 0 | 3.67 |
| 2 | 1.7K | 1 | 14.29 | 0 | 0.94 | 1 | 1 | 0 | 3.00 |
| 3 | 2.9K | 1 | 14.66 | 0 | 0.49 | 1 | 1 | 0 | 1.00 |
| 4 | 3.2K | 1 | 16.16 | 1 | 0.67 | 1 | 1 | 0 | 4.33 |
| 5 | 3.3K | 1 | 43.40 | 1 | 0.42 | 0 | 0 | 0 | 3.00 |
| 6 | 3.5K | 1 | 47.13 | 1 | 0.67 | 1 | 0 | 0 | 2.33 |
| 7 | 3.9K | 1 | 74.37 | 1 | 0.51 | 0 | 0 | 1 | 2.67 |
| 8 | 4.3K | 1 | 59.07 | 1 | 0.66 | 0 | 0 | 0 | 1.00 |
| 9 | 4.4K | 1 | 56.46 | 1 | 0.52 | 0 | 1 | 0 | 2.09 |
| 10 | 4.8K | 1 | 60.85 | 1 | 0.59 | 1 | 0 | 0 | 2.85 |
| 11 | 4.10K | 1 | 45.95 | 1 | 0.85 | 0 | 0 | 0 | 3.33 |
| 12 | 5.3K | 1 | 15.85 | 1 | 0.62 | 1 | 1 | 0 | 4.18 |
| 13 | 5.5K | 1 | 13.98 | 1 | 0.65 | 1 | 1 | 0 | 2.15 |
| 14 | 5.7K | 1 | 36.97 | 1 | 0.89 | 0 | 0 | 1 | 1.85 |
| 15 | 5.10K | 1 | 18.65 | 1 | 0.51 | 1 | 1 | 0 | 1.56 |
| 16 | 6.5K | 1 | 16.21 | 1 | 0.64 | 1 | 0 | 0 | 4.12 |
| 17 | 6.6K | 1 | 17.15 | 1 | 0.40 | 1 | 0 | 0 | 3.18 |
| 18 | 8.8K | 1 | 13.87 | 1 | 0.71 | 0 | 0 | 0 | 2.45 |
| 19 | 9.2K | 1 | 60.78 | 1 | 0.49 | 0 | 1 | 1 | 1.97 |
| 20 | 9.3K | 1 | 62.53 | 1 | 0.75 | 0 | 1 | 0 | 1.45 |
| 21 | 9.9K | 1 | 36.99 | 1 | 0.49 | 1 | 1 | 0 | 3.69 |
| 22 | 3.3H | 1 | 33.34 | 1 | 0.75 | 0 | 1 | 1 | 4.02 |
| 23 | 3.5H | 1 | 54.36 | 1 | 0.39 | 1 | 0 | 1 | 1.25 |
| 24 | 4.1H | 1 | 68.21 | 1 | 0.71 | 1 | 0 | 1 | 1.12 |
| 25 | 4.2H | 1 | 65.15 | 1 | 0.73 | 1 | 1 | 1 | 2.18 |
| 26 | 4.3H | 1 | 14.18 | 1 | 0.55 | 1 | 1 | 1 | 2.11 |
| 27 | 4.4H | 0 | 59.33 | 1 | 0.59 | 0 | 1 | 1 | 10.00 |
| 28 | 4.5H | 0 | 61.23 | 1 | 0.82 | 0 | 1 | 0 | 2.75 |
| 29 | 4.51H | 0 | 48.21 | 1 | 0.89 | 0 | 1 | 1 | 2.03 |
| 30 | 5.1H | 1 | 39.99 | 1 | 0.42 | 1 | 0 | 1 | 2.00 |
| 31 | 5.2H | 1 | 58.98 | 1 | 0.56 | 1 | 1 | 1 | 2.00 |
| 32 | 5.5H | 1 | 59.75 | 1 | 0.72 | 1 | 1 | 0 | 3.67 |
| 33 | 6.3H | 1 | 84.24 | 1 | 0.46 | 0 | 1 | 1 | 2.08 |
| 34 | 6.5H | 1 | 56.69 | 1 | 0.68 | 1 | 0 | 1 | 1.00 |
| 35 | 6.12H | 1 | 61.68 | 1 | 0.68 | 1 | 1 | 1 | 2.25 |
| 36 | 6.13H | 0 | 48.25 | 1 | 0.51 | 0 | 0 | 1 | 2.29 |
| 37 | 7.1H | 1 | 58.69 | 1 | 0.66 | 1 | 0 | 0 | 6.56 |
| 38 | 7.2H | 1 | 32.95 | 1 | 0.69 | 0 | 1 | 0 | 2.18 |
| 39 | 7.3H | 1 | 31.83 | 1 | 0.60 | 1 | 1 | 1 | 4.16 |
| 40 | 7.4H | 1 | 48.25 | 1 | 0.54 | 0 | 1 | 1 | 2.57 |
| 41 | 7.6H | 1 | 69.51 | 1 | 0.65 | 1 | 1 | 1 | 4.13 |
| 42 | 7.7H | 1 | 16.98 | 1 | 0.55 | 0 | 1 | 1 | 1.98 |
